# Physicians Are Responding Well

**Published:** 1918-09

**Authors:** 


### Physicians Are Responding Well.

---

Every once and awhile a statement is made that the doctors are not responding as they should to the call to the country's service. You have only to go into any city, town or village in the land and make inquiry to find that the doctors are responding by the thousands throughout the land. No war has ever called a greater proportion of physicians, because never before has the world appreciated the great help that they can render. In former wars it was expected that most of those wounded would die; now 95 per cent of the wounded are restored to the battle lines through the skill of the medical corps.

No profession has volunteered its services to the country more generously than the medical profession. About 24,000 physicians, or nearly one-fourth of the eligible physicians of the country, have expressed their willigness to resign practice and go to war. Some have already received their training and have gone to France. Some are waiting to be called, and about 2,000 are now in the camps receiving the special instruction that will fit them for a military life.

---

Medical Term.—"You must isolate the patient."

"All right, doctor; where shall we put the ice?"—*Baltimore American.*

